# Supplementary material for: Leaf Proteomic Analysis in Seedlings of Two Maize Landraces with Different Tolerance to Boron Toxicity
Source: Plants (Basel). 2023 Jun 15;12(12):2322. doi: 10.3390/plants12122322 (PMC10302400; doi:10.3390/plants12122322)
Supplement: Supplementary file 1 [file plants-12-02322-s001.zip › TABLE S3.pdf]

Supplementary table S3. Proteins with higher differential expression in Pachía leaves in response to boron (B) toxicity. This table shows the proteins that were strongly induced or repressed by B toxicity in Pachía by comparing their expressions with those of Pachía in medium with 0.05 mM B.

Protein ID[1]	Gene Name/ID[2]	Protein name/Annotation	FC[3]	*P*-Value[4]	Function/Biological process[5]
**AMINO ACID AND PEPTIDE METABOLISMS**					
**Proteins strongly induced by B toxicity in Pachía**					
B6SKB7	Zm00001d031013	Methylcrotonoyl-CoA carboxylase subunit alpha	4.44	0.0022	Leucine degradation
B6SWZ4	Zm00001d050336	Methylcrotonoyl-CoA carboxylase beta chain mitochondrial	2.85	0.0154	Leucine degradation
A0A1D6K836	Zm00001d029848	Branched-chain-amino-acid aminotransferase	2.35	0.0272	Branched-chain amino acid biosynthesis
B4G011	Zm00001d046923	D-3-phosphoglycerate dehydrogenase chloroplastic	2.31	0.0154	Serine biosynthesis
C4J411	Zm00001d028464	Imidazole glycerol phosphate synthase hisHF	2.17	0.0017	Histidine biosynthesis
C4JBG7	Zm00001d015088	3-isopropylmalate dehydratase large subunit	2.14	0.0320	Leucine biosynthesis
**Proteins strongly repressed by B toxicity in Pachía**					
B4FUH2	Zm00001d043382	Aspartate aminotransferase	0.48	0.0195	Amino acid metabolic process
B4FU01	Zm00001d045153	Cystathionine beta-lyase chloroplastic	0.44	0.0235	Methionine biosynthetic. Cysteine biosynthetic process via cystathionine
A0A1D6ICL3	Zm00001d021596	Adenosine 5-phosphosulfate reductase-like1	0.29	0.0140	Cysteine biosynthetic process. Sulfate reduction
B6TZD1	Zm00001eb168430	Methylthioribose-1-phosphate isomerase	0.24	0.0461	Methionine biosynthesis
**CARBON ASSIMILATION AND CALVIN CYCLE**					
**Proteins strongly induced by B toxicity in Pachía**					
A0A1D6FQE4	Zm00001d010321	Pyruvate phosphate dikinase	2.31	0.0449	C4 photosynthetic carbon assimilation cycle
**Proteins strongly repressed by B toxicity in Pachía**					
O24574	Zm00001d004894	Ribulose bisphosphate carboxylase small chain	0.38	0.0113	Carbon dioxide fixation
B4FQ59	Zm00001d017711	Phosphoribulokinase	0.33	0.0004	Calvin- Benson cycle
Q9ZT00	Zm00001eb164390	Ribulose bisphosphate carboxylase/oxygenase activase chloroplastic	0.26	0.0090	Carbon dioxide fixation. Rubisco activator activity
**CARBOHYDRATE METABOLISM**					
**Proteins strongly induced by B toxicity in Pachía**					
A0A1D6NE29	Zm00001d043662	Alpha-amylase 3 chloroplastic	2.05	0.0460	Starch degradation
**Proteins strongly repressed by B toxicity in Pachía**					
A0A1D6M7C2	Zm00001d038579	Phosphoglycerate kinase cytosolic	0.49	0.0136	Glycolysis and gluconeogenesis
B4FRC9	Zm00001d011965	Transaldolase	0.41	0.0407	Pentose-phosphate shunt
A0A1D6IJ76	Zm00001d022107	Glyceraldehyde-3-phosphate dehydrogenase A	0.34	0.0319	Carbon metabolism

**CELL DEATH**					
**Proteins strongly induced by B toxicity in Pachía**					
B4F8B9	Zm00001d018468	S-(hydroxymethyl)glutathione dehydrogenase	2.81	0.0027	Cell death. Formaldehyde oxidation (glutathione-dependent)
**CELL WALL**					
**Proteins strongly induced by B toxicity in Pachía**					
B4F9J1	Zm00001d046357	Beta-galactosidase	3.17	0.0092	Xyloglucan degradation
**DNA AND CHROMATIN ORGANIZATION AND DNA REPAIR**					
**Proteins strongly induced by B toxicity in Pachía**					
B6TGH8	Zm00001d034479	Histone H1	3.60	0.0349	Chromosome condensation. Nucleosome assembly. Nucleosome positioning
C0P6Q6	Zm00001d040416	DNA gyrase subunit B	3.48	0.0007	DNA topological change
**Proteins strongly repressed by B toxicity in Pachía**					
B6SK03	Zm00001d053295	Ubiquitin-conjugating enzyme E2 variant 1C	0.39	0.0409	DNA postreplication repair. Protein polyubiquitination
**LIPID METABOLISM**					
**Proteins strongly induced by B toxicity in Pachía**					
K7VQG5	Zm00001d008727	Phospholipase D	2.30	0.0244	Phospholipid degradation
A0A1D6NE81	Zm00001d043680	Phospholipase A1-IIdelta	2.02	0.0390	Lipid degradation
**Proteins strongly repressed by B toxicity in Pachía**					
B4FLS8	Zm00001d003584	12-oxo-phytodienoic acid reductase 5	0.33	0.0436	Fatty acid and oxylipin biosynthesis
**NITROGEN METABOLISM**					
**Proteins strongly induced by B toxicity in Pachía**					
A0A1D6PZA5	Zm00001d049995	Nitrate reductase	2.19	0.0077	Nitrate reductase (NADH) activity. Nitrate assimilation
**OTHERS**					
**Proteins strongly induced by B toxicity in Pachía**					
A0A1D6JGY3	Zm00001d026515	Molybdopterin molybdenumtransferase	2.92	0.0023	Molybdenum cofactor biosynthesis
A0A1D6HUN3	Zm00001d019040	D-2-hydroxyglutarate dehydrogenase mitochondrial	2.09	0.0380	Lysine degradation
**Proteins strongly repressed by B toxicity in Pachía**					
C0PDB6	Zm00001d039535	HXXXD-type acyl-transferase family protein	0.40	0.0112	N-acyltransferase activity
C0PE12	Zm00001d009877	Protein plastid transcriptionally active 16 chloroplastic	0.24	0.0121	Circadian rhythm
**OXIDATION AND REDUCTION PROCESSES**					
**Proteins strongly induced by B toxicity in Pachía**					
A0A1D6M498	Zm00001d038189	FAD/NAD(P)-binding oxidoreductase family protein	2.04	0.0101	Oxidoreductase activity
**PHOTOSYNTHETIC LIGHT REACTIONS**					

**Proteins strongly repressed by B toxicity in Pachía**					
B6SSB9	Zm00001d035859	Plastocyanin	0.50	0.0300	Photosynthetic electron transport
A0A1D6GU53	Zm00001d014564	Oxygen-evolving enhancer protein 1-1 chloroplastic	0.47	0.0268	Photosynthesis. Oxygen evolving activity. Photosystem II assembly and stabilization
B6SUC4	Zm00001d046786	Chlorophyll a-b binding protein, chloroplastic	0.41	0.0086	Photosynthesis. Light harvesting in photosystem I
B6T927	Zm00001d014349	NAD(P)H-quinone oxidoreductase subunit S chloroplastic (NDHS)	0.39	0.0095	Photosynthetic electron transport chain
P25709	NdhH	NAD(P)H-quinone oxidoreductase subunit H, chloroplastic	0.37	0.0022	Photosynthesis, light reaction. Photosynthetic electron transport chain. Couples the photosynthetic redox reaction to proton translocation
B6SP99	Zm00001d024148	Photosynthetic NDH subunit of subcomplex B 1 chloroplastic	0.33	0.0137	Photosynthetic electron transport in photosystem I
B4FJP7	Zm00001d027729	Photosynthetic NDH subunit of subcomplex B 2 chloroplastic	0.32	0.0169	Photosynthetic electron transport in photosystem I
B4FR80	Zm00001d033098	Post-illumination chlorophyll fluorescence increase (ZmPIFI)	0.28	0.0270	Chlororespiration
A0A1D6HS38	Zm00001d018779	Oxygen-evolving enhancer protein 2-1 chloroplastic (OEE2-1)	0.27	0.0110	Photosynthesis. Photosystem II oxygen evolving complex
B4FWG2	Zm00001d048422	Photosynthetic NDH subunit of subcomplex B 2 chloroplastic	0.25	0.0047	Photosynthetic electron transport flow around photosystem I to produce ATP
P19124	NdhJ	NAD(P)H-quinone oxidoreductase subunit J, chloroplastic	0.22	0.0147	Photosynthesis, light reaction, photosynthetic electron transport chain. Couples the photosynthetic redox reaction to proton translocation
A0A1X7YHG9	AtpA	ATP synthase subunit alpha (ATPα)	0.20	0.0166	Chloroplast ATP synthesis coupled proton transport
P46617	PetA	Cytochrome f	0.18	0.0193	Photosynthetic electron transport chain
P00827	Zm00001d009488	ATP synthase subunit beta, chloroplastic (ATPβ)	0.15	0.0076	Chloroplast ATP synthesis coupled proton transport
A0A1D6JYG6	Zm00001d028670	Photosynthetic NDH subunit of lumenal location 1 chloroplastic	0.13	0.0134	Part of photosystem II oxygen evolving complex
**PIGMENT BIOSYNTHESIS**					
**Proteins strongly repressed by B toxicity in Pachía**					
A0A1D6FAV8	Zm00001d008203	Protoporphyrinogen oxidase	0.38	0.0173	3,8-divinyl-chlorophyllide a and protoporphyrinogen IX biosynthesis
**PROTEIN DEGRADATION**					
**Proteins strongly induced by B toxicity in Pachía**					
B4FS65	Zm00001d005391	Cysteine protease 14	4.38	0.0146	Proteolysis. Proteolysis involved in protein catabolic process
A0A1D6HM49	Zm00001d018282	Subtilisin-like protease SBT1.4	3.70	0.0399	Serine protease. Serine-type endopeptidase activity. Proteolysis
A0A1D6H4R4	Zm00001d015962	Prolyl oligopeptidase family protein	3.58	0.0080	Proteolysis. Serine protease. Serine-type peptidase activity

Q84TL7	Zm00001d011036	Legumin-like protein	2.86	0.0453	Protein ubiquitination. Nutrient reservoir activity. Storage protein
A0A1D6KWW2	Zm00001d033194	Subtilisin-like protease	2.85	0.0403	Proteolysis. Serine protease. Serine-type endopeptidase activity
A0A1D6KV27	Zm00001d032956	Acylamino-acid-releasing enzyme	2.54	0.0086	Proteolysis. Serine protease. Serine-type endopeptidase activity
C0HI51	Zm00001d044102	Zn-dependent exopeptidase superfamily protein	2.53	0.0131	Proteolysis. Aminopeptidase. Metalloaminopeptidase activity
Q84TL6	Zm00001d035597	Legumin-like protein	2.36	0.0386	Protein ubiquitination. Storage protein. Nutrient reservoir activity
A0A1D6HL34	Zm00001d018145	Presequence protease 2 chloroplastic/mitochondrial	2.22	0.0180	Proteolysis. Metalloendopeptidase activity. Protein processing
K7VGG8	Zm00001d010522	ATP-dependent zinc metalloprotease FTSH 10 mitochondrial	2.07	0.0359	Proteolysis. Metalloprotease mitochondrial
C4JC43	Zm00001d049100	Target of Myb protein 1	2.04	0.0450	Proteolysis. Protein transport to vacuole involved in ubiquitin-dependent protein catabolic process via the multivesicular body sorting pathway
**Proteins strongly repressed by B toxicity in Pachía**					
A0A1D6H558	Zm00001d016036	Chloroplast processing peptidase	0.47	0.0438	Protease. Serine-type endopeptidase activity
B4FQJ6	Zm00001d018309	26S protease regulatory subunit 7 homolog A	0.46	0.0249	Proteolysis. Protein catabolic process. Peptidase activity
A0A1D6FKP2	Zm00001d009613	Protease Do-like 1 chloroplastic	0.45	0.0496	Proteolysis. Serine-type endopeptidase activity
K7TTX0	Zm00001d025628	Plant UBX domain-containing protein 4	0.44	0.0107	Proteasome-mediated ubiquitin-dependent protein catabolic process
**PROTEIN STABILIZATION AND FOLDING**					
**Proteins strongly induced by B toxicity in Pachía**					
A0A1D6FN98	Zm00001d009948	Heat shock 70 kDa protein 14	2.28	0.0487	Protein folding. Stress response
B6SZ69	Zm00001d028630	Heat shock cognate 70 kDa protein 2	2.02	0.0398	Protein refolding. Stress response
**Proteins strongly repressed by B toxicity in Pachía**					
A0A1D6KC46	Zm00001d030346	Hsp20/alpha crystallin family protein	0.49	0.0499	Chaperone. Response to heat
C0PKD9	Zm00001d052101	Chaperonin10	0.42	0.0428	Chaperone cofactor-dependent protein refolding Protein folding. Chaperone
G2XK63	Zm00001d040257	T-complex protein 1 subunit beta	0.27	0.0065	Protein folding. Chaperone
B4FR04	Zm00001d019052	Peptidylprolyl isomerase	0.23	0.0205	Protein folding. Rotamase
**REACTIVE OXYGEN SPECIES (ROS) SCAVENGING PATHWAYS / RESPONSE TO OXIDATIVE STRESS**					
**Proteins strongly induced by B toxicity in Pachía**					
A0A1D6K5D2	Zm00001d029457	Nucleoredoxin1	2.91	0.0117	Protection against oxidative stress. Cellular oxidant detoxification
A0A1D6MSE3	Zm00001d040721	Dihydrolipoyl dehydrogenase	2.30	0.0273	Cell redox homeostasis

A0A1D6JPH3	Zm00001d027769	Glutathione reductase	2.21	0.0053	Cell redox homeostasis. Cellular oxidant detoxification. Glutathione metabolic process.
K7US39	Zm00001d009163	Dihydrolipoyl dehydrogenase	2.19	0.0088	Cell redox homeostasis
**RIBOSOME BIOGENESIS**					
**Proteins strongly induced by B toxicity in Pachía**					
B4FPB7	Zm00001d006100	60S ribosomal protein L7a	2.63	0.0051	Ribosome biogenesis. Maturation of LSU-rRNA
K7UTH7	Zm00001d009596	GTPase ERA1 chloroplastic	2.61	0.0108	Ribosome biogenesis. Ribosomal small subunit assembly. rRNA processing
B4F7Y1	Zm00001d031640	60S ribosomal protein L7a-1	2.39	0.0448	Ribosomal protein. Maturation of LSU-rRNA
**RNA BINDING AND PROCESSING**					
**Proteins strongly induced by B toxicity in Pachía**					
A0A1D6HT50	Zm00001d018891	Chloroplast RNA processing 4	2.60	0.0142	mRNA catabolic process
**SIGNALING**					
**Proteins strongly repressed by B toxicity in Pachía**					
P49235	Zm00001eb411380	4-hydroxy-7-methoxy-3-oxo-3,4-dihydro-2H-1,4-benzoxazin-2-yl glucoside beta-D-glucosidase 1, chloroplastic	0.19	0.0090	Cytokinin signaling pathway

**STRESS**					
**Proteins strongly induced by B toxicity in Pachía**					
B4F9K2	Zm00001d005315	Calcium-dependent lipid-binding (CaLB domain) family protein	2.11	0.0402	Defense response. Response to stress
**TRANSCRIPTION AND TRANSLATION PROCESSES**					
**Proteins strongly induced by B toxicity in Pachía**					
A0A1D6LEN8	Zm00001d035139	MA3 domain-containing protein	4.95	0.0073	Negative regulation of transcription, DNA-templated. Regulation of translation
Q6R9D1	GRMZM5G806488	Ribosomal protein S7	3.89	0.0202	Translation. Ribosomal small subunit assembly. Structural constituent of ribosome
A0A1D6IAN8	Zm00001d021400	Octicosapeptide/Phox/Bem1p (PB1) domain-containing protein / tetratricopeptide repeat (TPR)-containing protein	3.47	0.0323	RNA processing
C0P456	Zm00001d002789	Pentatricopeptide repeat-containing protein	3.26	0.0259	Likely involved in posttranscriptional control of gene expression in organelles
A0A1D6NR59	Zm00001d044745	Probable alanine--tRNA ligase, chloroplastic	2.74	0.0097	Translation. Alanyl-tRNA aminoacylation
A0A1D6LIV5	Zm00001d035802	Phenylalanine--tRNA ligase beta subunit cytoplasmic	2.56	0.0314	Translation. Phenylalanyl-tRNA aminoacylation
B6T5F2	Zm00001d011992	60S ribosomal protein L13	2.48	0.0387	Translation. Structural constituent of ribosome
A0A1D6HM03	Zm00001d018274	Isoleucine--tRNA ligase chloroplastic/mitochondrial	2.29	0.0087	Translation. Isoleucyl-tRNA aminoacylation
A0A1D6QAN9	Zm00001d051885	ATG8-interacting protein 1	2.28	0.0047	Box C/D RNA 3'-end processing. rRNA processing
A0A1D6FRP3	Zm00001d010530	Cysteine--tRNA ligase 1 cytoplasmic	2.26	0.0470	Translation. Cysteinyl-tRNA aminoacylation

B4FMD3	Zm00001d012978	40S ribosomal protein S23-2	2.13	0.0451	Translation. Structural constituent of ribosome
K7UTZ2	Zm00001d009761	Spliceosome RNA helicase BAT1 isoform 1	2.10	0.0455	RNA splicing. RNA helicase activity
K7TY03	Zm00001d023741	Alanine--tRNA ligase	2.07	0.0144	Translation. Alanyl-tRNA aminoacylation
B4FYR2	Zm00001d038865	60S ribosomal protein L28	2.05	0.0275	Translation. Structural constituent of ribosome
B6U151	Zm00001d002104	Glutamyl-tRNA(Gln) amidotransferase subunit A, chloroplastic/mitochondrial	2.02	0.0464	Mitochondrial translation
**Proteins strongly repressed by B toxicity in Pachía**					
C0P7X7	Zm00001d034808	30S ribosomal protein S6 alpha chloroplastic	0.50	0.0059	Translation. Structural constituent of ribosome
B4FUZ5	Zm00001d047581	30S ribosomal protein S1	0.46	0.0055	Translation. Ribosomal protein
O50018	Zm00001d046449	Elongation factor 1-alpha	0.29	0.0269	Translation. Translation elongation factor activity
**TRANSPORTERS AND TRANSPORT PROCESSES**					
**Proteins strongly induced by B toxicity in Pachía**					
B6SP43	Zm00001d007597	ABC family1	4.54	0.0103	ATPase-coupled transmembrane transporter activity
A0A1D6H2R4	Zm00001d015569	H^+-exporting diphosphatase	4.34	0.0050	Ion transport. Pyrophosphate hydrolysis-driven proton transmembrane transporter activity
A0A1D6MS70	Zm00001d040686	Protein translocase subunit SECA1 chloroplastic	4.12	0.0173	Protein transport
A0A1D6DSW6	Zm00001d001788	K^+ efflux antiporter 2 chloroplastic	3.79	0.0414	Chloroplast potassium ion transport
B6T5R1	Zm00001d010504	Ran-binding protein 1	3.16	0.0492	Intracellular transport. Protein and mRNA transport. Nucleocytoplasmic transport
A0A1D6KSB0	Zm00001d032615	Protein TIC110 chloroplastic	2.35	0.0118	Protein import into chloroplast stroma
**NOT WELL-KNOWN PROTEINS**					
**Proteins strongly induced by B toxicity in Pachía**					
A0A1D6KKK1	Zm00001d031677	MtN19-like protein	2.62	0.0464	Not well determined
A0A1D6JI62	Zm00001d026632	Stem-specific protein TSJT1	2.43	0.0283	Not well determined

Only proteins considered differentially expressed, namely those with fold-changes ≥2.0 or ≤ 0.5 and *P*-values ≤ 0.05, are shown in this table. Strongly induced proteins are highlighted with light green rows and strongly repressed proteins with light red rows.

[1]Protein ID: protein identification number in the UniProt database. [2]Gene Name: name or ID number of the corresponding gene of the differentially expressed protein as searched in the Maize Genetics and Genomics Database (MaizeGDB; https://www.maizegdb.org/. Accessed between June 6, 2022 and January 24, 2023). [3]Fold Change is expressed as the ratio of LFQ intensities (on a logarithmic scale) of proteins between 10 and 0.05 mM B treatments in Pachía. Results were obtained from 3-4 separate plants. [4]*p*-value: statistical level (using Student's *t*-test) below ≤0.05, at which differential protein expression was accepted as significant. [5]Function/Biological process: annotated biological functions or biological process based on different databases. For more details, see Materials and Methods.
