# Supplementary material for: Leaf Proteomic Analysis in Seedlings of Two Maize Landraces with Different Tolerance to Boron Toxicity
Source: Plants (Basel). 2023 Jun 15;12(12):2322. doi: 10.3390/plants12122322 (PMC10302400; doi:10.3390/plants12122322)
Supplement: Supplementary file 1 [file plants-12-02322-s001.zip › TABLE S4.pdf]

Supplementary table S4. Proteins with higher differential expression in Sama leaves in response to boron (B) toxicity. This table shows the proteins that were strongly induced or repressed by B toxicity in Sama by comparing their expressions with those of Sama in medium with 0.05 mM B.

Protein ID[1]	Gene Name/ID[2]	Protein name/Annotation	FC[3]	*P*-value[4]	Function/Biological process[5]
**AMINO ACID AND PEPTIDE METABOLISMS**					
**Proteins strongly induced by B toxicity in Sama**					
B6SKB7	Zm00001d031013	Methylcrotonoyl-CoA carboxylase subunit alpha	3.56	0.0049	Leucine degradation
C4J3S6	Zm00001d004960	2-isopropylmalate synthase 1 chloroplastic	2.17	0.0025	Leucine biosynthesis
**CARBON ASSIMILATION AND CALVIN CYCLE**					
**Proteins strongly repressed by B toxicity in Sama**					
O24574	Zm00001d004894	Ribulose bisphosphate carboxylase small chain	0.33	0.0466	Carbon dioxide fixation
P05348	Rbcs	Ribulose bisphosphate carboxylase small chain, chloroplastic	0.13	0.0096	Carbon dioxide fixation
**CELL DIVISION**					
**Proteins strongly induced by B toxicity in Sama**					
C0P4T2	Zm00001d042664	Patellin-1	3.05	0.0149	Cell division and cell cycle
**NUCLEOTIDE, PURINE AND PYRIMIDINE METABOLISM**					
**Proteins strongly repressed by B toxicity in Sama**					
A0A1D6P7V2	Zm00001d047217	5-hydroxyisourate hydrolase	0.50	0.0136	Purine metabolism
**PHOTOSYNTHETIC LIGHT REACTIONS**					
**Proteins strongly repressed by B toxicity in Sama**					
A0A1D6HS38	Zm00001d018779	Oxygen-evolving enhancer protein 2-1 chloroplastic (OEE2-1)	0.48	0.0354	Photosynthesis. Photosystem II oxygen evolving complex
B6SSN3	Zm00001d015385	Chlorophyll a-b binding protein, chloroplastic	0.43	0.0435	Light harvesting in photosystem I
B4FWG2	Zm00001d048422	Photosynthetic NDH subunit of subcomplex B 2 chloroplastic	0.41	0.0200	Photosynthetic electron transport flow around photosystem I to produce ATP
P06670	NdhK	NAD(P)H-quinone oxidoreductase subunit K, chloroplastic	0.38	0.0149	Photosynthetic electron transport coupled photosynthetic proton transport
A0A1X7YHF7	PsbD	Photosystem II D2 protein	0.35	0.0347	Photosynthetic electron transport in photosystem II
P46617	PetA	Cytochrome f	0.29	0.0161	Photosynthetic electron transport chain
B6SQV5	Zm00001d049387	Photosystem II 10 kDa polypeptide	0.14	0.0438	Photosynthesis. Photosystem II oxygen evolving complex
**PROTEIN STABILIZATION AND FOLDING**					
**Proteins strongly repressed by B toxicity in Sama**					
A0A1D6GJM6	Zm00001d013455	Peptidylprolyl isomerase	0.40	0.0275	Protein folding. Rotamase

C4J6Y2	Zm00001d018077	Peptidylprolyl isomerase	0.18	0.0422	Protein folding. Rotamase
**REACTIVE OXYGEN SPECIES (ROS) SCAVENGING PATHWAYS / RESPONSE TO OXIDATIVE STRESS**					
**Proteins strongly induced by B toxicity in Sama**					
B4FSM5	Zm00001d040341	Peroxiredoxin	2.68	0.0112	Cellular response to oxidative stress. Hydrogen peroxide catabolic process. Cell redox homeostasis
**Proteins strongly repressed by B toxicity in Sama**					
B6U038	Zm00001d005482	Thiol-disulfide isomerase and thioredoxins	0.44	0.0223	Antioxidant activity. Cellular oxidant detoxification. Thioredoxin-dependent peroxiredoxin activity
B4FZ35	Zm00001d002240	CHL-*Zea mays* Chloroplastic lipocalin	0.31	0.0272	Response to oxidative stress. Violaxanthin, antheraxanthin and zeaxanthin interconversion
**SECONDARY METABOLISM**					
**Proteins strongly induced by B toxicity in Sama**					
O64411	Zm00001d024281	Polyamine oxidase 1 (PAO1)	3.34	0.0108	Spermine degradation. Amine and polyamine degradation
**Proteins strongly repressed by B toxicity in Sama**					
B6TAE7	Zm00001d028575	Tropinone reductase	0.44	0.0313	Tropane alkaloid biosynthesis
**STRESS**					
**Proteins strongly induced by B toxicity in Sama**					
A0A1D6NJS4	Zm00001d044222	Tetratricopeptide repeat (TPR)-containing protein	2.12	0.0428	N-terminal peptidyl-methionine acetylation. Protein maturation
**TRANSCRIPTION AND TRANSLATION PROCESSES**					
**Proteins strongly induced by B toxicity in Sama**					
A0A1D6IBP5	Zm00001d021507	Asparagine--tRNA ligase chloroplastic/mitochondrial	2.66	0.0491	Translation. Asparaginyl-tRNA aminoacylation
B4FSE0	Zm00001d033913	Alba DNA/RNA-binding protein	2.48	0.0244	Translational initiation. RNA binding
B6T872	Zm00001d021020	60S ribosomal protein L32	2.28	0.0415	Translation. Structural constituent of ribosome
A0A1D6LIV5	Zm00001d035802	Phenylalanine--tRNA ligase beta subunit cytoplasmic	2.23	0.0093	Translation. Phenylalanyl-tRNA aminoacylation
B4FJ27	Zm00001d011741	40S ribosomal protein S24	2.13	0.0363	Translation. Structural constituent of ribosome
**TRANSPORTERS AND TRANSPORT PROCESSES**					
**Proteins strongly induced by B toxicity in Sama**					
B6SP43	Zm00001d007597	ABC family1	2.69	0.0125	ATPase-coupled transmembrane transporter activity

Only proteins considered differentially expressed namely those with fold-changes ≥2.0 or ≤ 0.5 and *P*-values ≤ 0.05, are shown in this table. Strongly induced proteins are highlighted with light green rows and strongly repressed proteins with light red rows.

[1]Protein ID: protein identification number in the UniProt database. [2]Gene Name: name or ID number of the corresponding gene of the differentially expressed protein as searched in the Maize Genetics and Genomics Database (MaizeGDB; https://www.maizegdb.org/. Accessed between June 6, 2022 and January 24, 2023). [3]Fold Change is expressed as the ratio of LFQ intensities (on a logarithmic scale) of proteins between 10 and 0.05 mM B treatments in Sama. Results were obtained from 3-4 separate plants. [4]*p*-value: statistical level (using Student's *t*-test), at which differential protein expression was accepted as significant (≤0.05). [5] Function/Biological process: annotated biological functions or biological process based on different databases. For more details, see Materials and Methods.
